# Equipping providers to offer novel MPTs: Developing counseling messages for the Dual Prevention Pill in clinical studies and beyond

**DOI:** 10.3389/frph.2023.1155948

**Published:** 2023-05-22

**Authors:** Kate Segal, Danielle M. Harris, Andy Carmone, Lisa B. Haddad, Sanjay Hadigal, Karin Hatzold, Chris Jones, Eva Lathrop, Jennifer Mason, Meridith Mikulich

**Affiliations:** ^1^ AVAC, Product Introduction and Access, New York, NY, United States; ^2^ Catalyst Global, Carlsbad, CA, United States; ^3^ Clinton Health Access Initiative (CHAI), Boston, MA, United States; ^4^ Population Council, Center for Biomedical Research, New York, NY, United States; ^5^ Viatris, Department of Global Medical Affairs, Pittsburgh, PA, United States; ^6^ Population Services International (PSI), Washington, DC, United States; ^7^ Mann Global Health, Columbus, NC, United States; ^8^ United States Agency for International Development (USAID), Office of Population and Reproductive Health, Bureau of Global Health, Washington, DC, United States

**Keywords:** pre-exposure prophlyaxis, oral contraception, HIV prevention, family planning, sexual and reproductive health, multi-purpose prevention technologies, service delivery, provider counseling

## Abstract

**Introduction:**

The pipeline for multi-purpose prevention technologies includes products that simultaneously prevent HIV, pregnancy and/or other sexually transmitted infections. Among these, the Dual Prevention Pill (DPP) is a daily pill co-formulating oral pre-exposure prophylaxis (PrEP), and combined oral contraception (COC). Clinical cross-over acceptability studies for the DPP require training providers to counsel on a combined product. From February 2021–April 2022, a working group of eight HIV and FP experts with clinical and implementation expertise developed counseling recommendations for the DPP based on existing PrEP/COC guidance.

**Assessment of policy/guidelines options and implications:**

The working group conducted a mapping of counseling messages from COC and oral PrEP guidance and provider training materials. Six topics were prioritized: uptake, missed pills, side effects, discontinuation and switching, drug interactions and monitoring. Additional evidence and experts were consulted to answer outstanding questions and counseling recommendations for the DPP were developed. *Missed pills* was the topic with the most complexity, raising questions about whether women could “double up” on missed pills or skip the last week of the pack to recover protection faster. *Uptake* required aligning the time to reach protective levels for both DPP components and explaining the need to take DPP pills during week 4 of the pack. The potential intensity of DPP *side effects*, given the combination of oral PrEP with COC, was an important consideration. *Discontinuation and switching* looked at managing risk of HIV and unintended pregnancy when stopping or switching from the DPP. Guidance on *drug interactions* contended with differing contraindications for COC and PrEP. *Monitoring* required balancing clinical requirements with potential user burden.

**Actionable recommendations:**

The working group developed counseling recommendations for the DPP to be tested in clinical acceptability studies. *Uptake*: Take one pill every day for the DPP until the pack is empty. Days 1–21 contain COC and oral PrEP. Days 22–28 do not contain COC to allow for monthly bleeding, but do contain oral PrEP and pills should be taken to maintain HIV protection. Take the DPP for 7 consecutive days to reach protective levels against pregnancy and HIV. *Missed pills*: If you miss 1 pill multiple times in a month or 2+ consecutive pills, take the DPP as soon as you remember. Do not take more than 2 pills in a day. If 2+ consecutive pills are missed, only take the last missed pill and discard the other missed pills. *Side effects*: You may experience side effects when you start using the DPP, including changes to monthly bleeding. Side effects are typically mild and go away without treatment. *Discontinuation/switching*: If you decide to discontinue use of the DPP, but want to be protected from HIV and/or unintended pregnancy, in most cases, you can begin using PrEP or another contraceptive method right away. *Drug interactions*: There are no drug-drug interactions from combining oral PrEP and COC in the DPP. Certain medications are not recommended due to their contraindication with oral PrEP or COC. *Monitoring*: You will need to get an HIV test prior to initiating or restarting the DPP, and every 3 months during DPP use. Your provider may recommend other screening or testing.

**Discussion:**

Developing recommendations for the DPP as a novel MPT posed unique challenges, with implications for efficacy, cost, and user and provider comprehension and burden. Incorporating counseling recommendations into clinical cross-over acceptability studies allows for real-time feedback from providers and users. Supporting women with information to use the DPP correctly and confidently is critically important for eventual scale and commercialization.

## 1. Introduction

Despite dedicated efforts to reduce unmet need for family planning (FP) and HIV incidence globally, cisgender women encounter barriers to accessing contraception and HIV prevention in many settings, hindering progress toward global targets. Across sub-Saharan Africa, women of reproductive age account for 65% of new HIV infections among adults ages 15–49 and have varying levels of unmet need for FP, ranging from 8% in Botswana to 27% in Angola among all women of reproductive age (1, 2). Adolescent girls and young women (AGYW) ages 15–24 bear a greater HIV disease burden, comprising a staggering 77% of new infections among young people and nearly half among women ages 15–49 in the region (1). Globally, AGYW also have the highest levels of unmet need for FP (3). Oral pre-exposure prophylaxis (PrEP) can be a highly effective daily antiretroviral (ARV)-based pill for HIV prevention, but uptake, continuation and effective use by cisgender women has lagged since its approval by the U.S. Food & Drug Administration (FDA) in 2012 (4). Daily pill-taking remains a challenge for many women, who may require discretion to be able to use PrEP, as intimate partner violence can contribute to low rates of continued use, among other factors (5, 6). Though uptake of oral PrEP in sub-Saharan Africa has rapidly grown since 2020, and two additional PrEP options—the dapivirine vaginal ring (PrEP ring) and injectable cabotegravir (CAB for PrEP)—have been approved in several African countries (7), programs have been slow to integrate FP and HIV prevention services despite the layered sexual and reproductive health (SRH) needs of women and girls (8).

Within this context, multi-purpose prevention technologies (MPTs) that simultaneously prevent pregnancy, HIV and/or other sexually transmitted infections (STIs) could address persistent shortcomings in women's access to comprehensive SRH services. Multiple discrete choice experiments have found that women and heterosexual couples prefer MPTs to single-indication HIV prevention products and prefer novel MPT formulations to male condoms (9, 10). One modeling study in South Africa estimated that MPTs preventing both pregnancy and HIV could quadruple demand for HIV prevention products among adolescent girls compared to products that consist solely of HIV prevention medication (11). Growing investment in MPT research and development (R&D) (12) signals the potential for MPTs to transform the prevention field: the MPT pipeline contains 28 products with diverse delivery forms and formulations as of December 2022, including oral tablets, intravaginal rings, injectables and implants (13).

Among these, the Dual Prevention Pill (DPP) is a daily pill co-formulating tenofovir disoproxil and emtricitabine (TDF/FTC), the only approved formulation of oral PrEP for cisgender women, and levonorgestrel and ethinyl estradiol (LNG/EE), a combined oral contraceptive (COC). While the majority of MPTs in development are in the pre-clinical phase, the DPP only requires a bioequivalence study to demonstrate that its drug components are bioequivalent in combination compared to oral PrEP and COC taken separately (14). With this streamlined regulatory process, the DPP under development is likely to complete FDA regulatory requirements for approval and could reach the market in 2024. Pending regulatory approval, the DPP will be the next MPT to market and the only MPT alternative to male and female condoms for the foreseeable future.

Clinical cross-over acceptability studies for the DPP in South Africa (15) (*n* = 96) and Zimbabwe (16) (*n* = 30) are currently evaluating adherence, acceptability and preference for a single, over-encapsulated DPP compared to two separate PrEP and COC tablets in cisgender women ages 16–40 who are interested in using an HIV prevention product in combination with a contraceptive method. These studies require providers to counsel participants on a novel MPT, particularly on instructions for use, which will differ from counseling on separate oral PrEP and COC products (17). FP providers are well-versed in counseling on voluntarism and informed choice, where they explain the risks and benefits of available methods and support clients to choose and use the method they prefer (18). They have expressed that training on PrEP and HIV prevention services would build their confidence to deliver them alongside FP (8). Yet due to long-siloed FP and HIV services (8), approaches to counseling for and delivery of HIV and FP are different, and integrated services are not consistently or uniformly provided, which could slow down rollout of novel MPTs like the DPP in places where women would be more likely to access it.

Reconciling counseling guidance for oral PrEP and COC is needed to develop guidelines and training materials for providers to counsel on the DPP in clinical cross-over acceptability studies and for future DPP service delivery. Lessons from delivering the DPP in these studies can inform provider counseling approaches to offering comprehensive PrEP and FP options in real-world settings, including the DPP and future MPTs. In February 2021, a working group of eight experts across the FP/SRH and HIV disciplines, with clinical expertise and implementation experience, was assembled. Working group members came from product developers, implementing partners, research organizations and development agencies, the majority of which are also involved in planning for the introduction of the DPP. From February 2021–April 2022, this working group developed counseling recommendations for the DPP based on existing oral PrEP and COC guidance to inform provider counseling in DPP acceptability studies.

## 2. Assessment of policy/guidelines options and implications

### 2.1. Methodology

To develop counseling recommendations for the DPP, the working group conducted a mapping of counseling messages from existing COC and oral PrEP guidance and relevant provider training materials, recognizing that the DPP is not yet available for use. Several assumptions were agreed upon to guide inclusion criteria. Counseling recommendations would focus specifically on the DPP as a novel product, and on counseling areas most in need of reconciliation between oral PrEP and COC. Recommendations would presume that the client has already received informed choice counseling on the full range of HIV prevention and contraceptive methods available and has selected the DPP. Ideally, comprehensive counseling covering broader SRH issues such as gender-based violence will have also been performed, but was outside the scope of this analysis. In addition, DPP counseling recommendations assume the client meets medical eligibility requirements for both the COC and oral PrEP components of the DPP, including FP screening and a negative HIV test. Lastly, recommendations were developed based on the understanding that the DPP will follow a 28-day regimen, with three weeks of co-formulated PrEP/COC pills followed by one week of PrEP pills only, which has important implications for some counseling topics. Of note, the terms “cisgender women” and “women” are used throughout this manuscript to be consistent with the study population in DPP clinical cross-over acceptability studies, and to distinguish this population from prevention literature and guidance pertaining to cisgender men. The authors recognize that communities are gender-diverse, and that some people for whom DPP may be an option would not identify or be categorized as cisgender women.

Eleven PrEP and COC counseling guidance materials and tools were included in the initial mapping (19–29) (Table 1). The World Health Organization (WHO) was the primary source of information for guidance on oral PrEP and COC. Once materials for inclusion were selected, relevant information was categorized by counseling topic. For each topic, we distilled where oral PrEP and COC guidance converged and diverged. We then identified outstanding questions and core elements to address in counseling for the DPP. Six topics were prioritized based on clinical relevance for DPP acceptability studies: (1) uptake, (2) missed pills, (3) side effects, (4) discontinuation and switching, (5) drug interactions and (6) monitoring.

**Table 1:** Primary counseling guidance materials and tools consulted for oral PrEP and COC.

Product	Author	Title	Chapter/Module
Oral PrEP	WHO	Implementation Tool for Pre-Exposure Prophylaxis of HIV Infection	Module 1: Clinical
			Module 10: Testing Providers
			Module 11: PrEP Users
			Module 12: Adolescents and Young Adults
	WHO	Consolidated guidelines on HIV prevention, testing, treatment, service delivery and monitoring: recommendations for a public health approach (2021)	N/A
	WHO	Differentiated and simplified pre-exposure prophylaxis for HIV prevention: update to WHO implementation guidance. Technical Brief	N/A
	OPTIONS Consortium	Provider Training Package: Effective Delivery of Oral PrEP for Adolescent Girls and Young Women	N/A
	Southern African HIV Clinicians Society	PrEP Training Curriculum in Southern Africa	N/A
	FHI 360	Guidance on Providing Informed-Choice Counseling on Sexual Health for Women Interested in PrEP	N/A
COC	WHO	Family Planning: A Global Handbook for Providers	Providing Combined Oral Contraceptives
	Population Council	Balanced Counseling Strategy Plus (BCS+)	Counseling Cards
			Method Brochures
	Population Services International	Counseling for Choice (C4C): The Choice Book for Providers	N/A

Additional literature and subject matter experts were consulted outside of the sources included in the initial mapping to answer specific outstanding questions. These include WHO's updated HIV guidelines, which were released after the initial mapping was completed (30). Subject matter experts from research institutions provided supplementary contextual information about available literature and their perspectives on gaps in data, namely on oral PrEP toxicity and missed pills guidance (see Acknowledgments for more information). Counseling recommendations for the DPP were developed from all available information and refined based on working group discussions and consensus.

Preliminary counseling recommendations were reviewed by researchers at the Population Council, sponsors for the DPP clinical cross-over acceptability studies, and Wits Reproductive Health and HIV Institute, implementing partner for the DPP acceptability study in South Africa. Acceptability study protocols were adapted to reflect counseling messages for the DPP developed by the working group.

### 2.2. Analysis

#### 2.2.1. Missed pills

**Figure 1:**
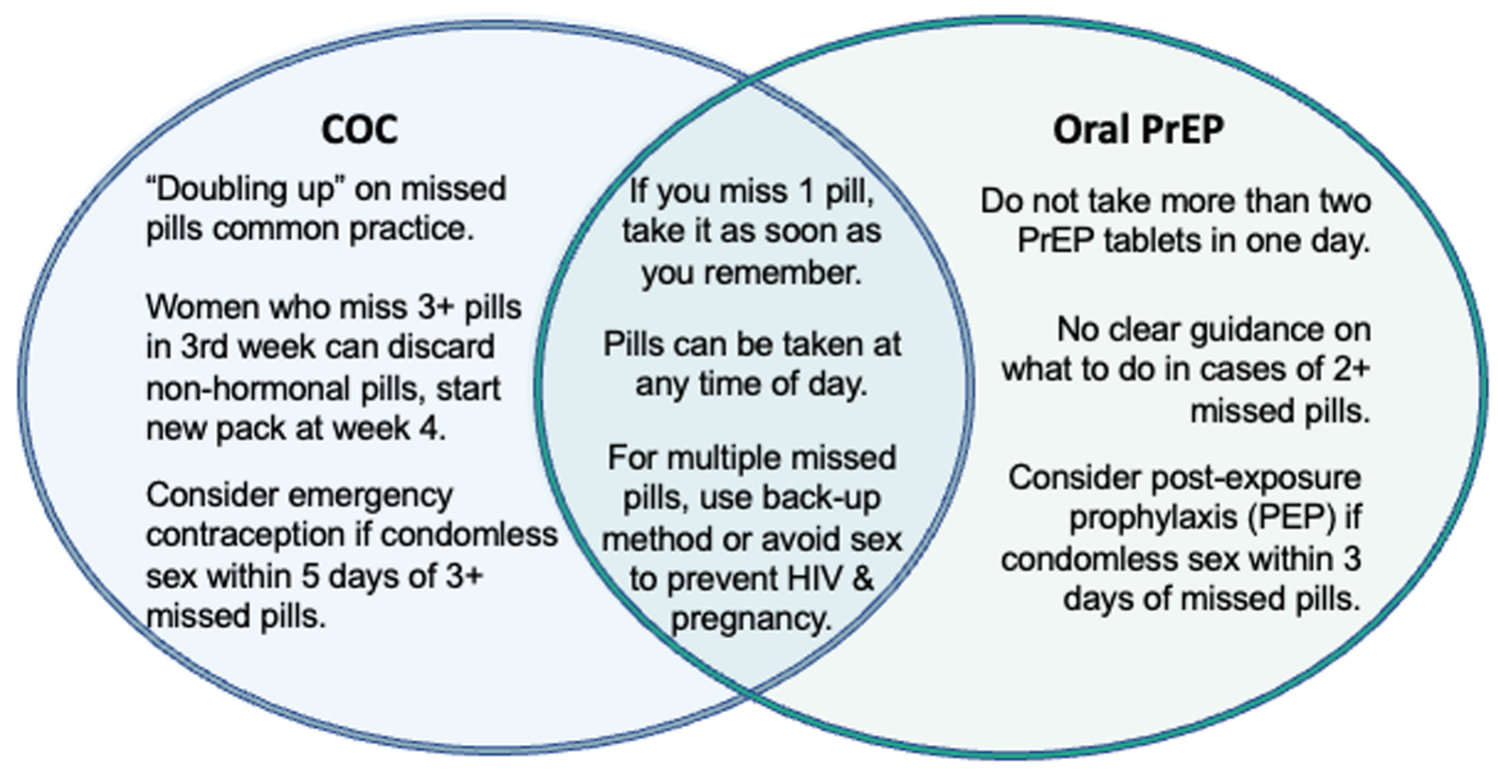
Illustrative example of summarized COC and oral PrEP missed pills guidance.

Missed pills was the counseling topic requiring the most attention when reconciling oral PrEP and COC guidance, as it had greater points of divergence in guidance documents and real-world implementation than the other five topics included in the mapping (Figure 1). COC and oral PrEP guidance on missed pills overlap in a few areas, stipulating that: pills can be taken at any time of day; in the case of one missed pill, the client should take the missed dose as soon as they remember; and in the case of multiple missed pills, the client should consider using a back-up method or avoiding sex to prevent HIV and pregnancy.

However, COC and oral PrEP guidance and practice diverge for counseling on multiple missed pills (defined as 1 missed pill multiple times in a month or 2+ consecutive missed pills), primarily pertaining to the quantity and timing of missed pills in a month. For COCs, taking two pills at a time in the event of a missed dose, or “doubling up,” is common practice and can be done multiple times throughout the month. By contrast, WHO guidance permits “occasional” doubling up on oral PrEP and advises against taking more than two PrEP tablets in one day. The guidance is unclear on how clients should proceed in cases of 2+ missed oral PrEP pills (21), and there is limited published evidence on toxicity of multiple PrEP doses in cisgender women. Differences in quantities of missed pills have implications for recommending use of a back-up method: COC users are counseled to consider emergency contraception (EC) if they have condomless sex within five days of 3+ missed pills, while oral PrEP users are advised to consider post-exposure prophylaxis (PEP) if they have condomless sex within three days of missed pills, with no quantity of missed pills specified. The working group considered the feasibility of aligning recommendations for taking EC and PEP in cases of multiple missed DPP pills to simplify counseling messages for users.

Assessing whether and how often clients could reasonably double up on the DPP, particularly given the paucity of data on oral PrEP toxicity in cisgender women, required additional desk research and expert consultation. Most studies with findings on double-dosing of oral PrEP have only been conducted with cisgender men who have sex with men (31, 32), leading WHO to recommend event-driven PrEP for this population in its guidelines (30). Recent research suggests that a single double-dose of oral PrEP pills in women is safe and would only increase protection back to a steady-state level in the event of a missed pill (33, 34).

**Figure 2:**
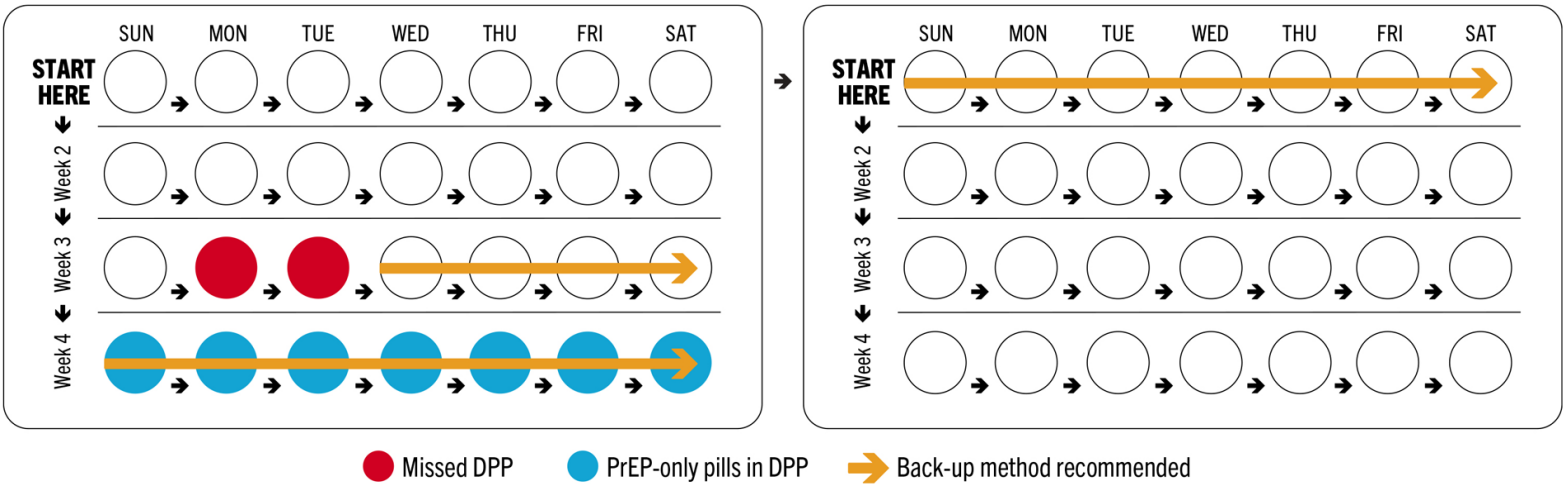
Illustration of timeline to recover pregnancy protection after multiple missed DPP pills.

Timing of missed pills is particularly critical for COC users in light of two considerations: (1) 7 consecutive days of COC use are required to reach protective levels against pregnancy (26) and (2) in a 28-day COC pack, the last week (week 4, or days 22–28) contains non-hormonal pills, or placebos. As such, missing multiple COC pills in weeks 1 and 3, which are the weeks that follow and precede week 4, respectively, could increase risk of unintended pregnancy by extending the period of time without hormonal pills beyond what is recommended (Figure 2). COC guidance permits a client who misses 3+ pills in week 3 of the month to discard the remaining pills and instead begin a new pack, enabling them to recoup pregnancy protection faster. Otherwise, use of a back-up method is recommended until the client has taken COCs for 7 consecutive days.

Conversely, all oral PrEP pills in a monthly regimen contain the same active pharmaceutical ingredients (APIs); therefore, timing of missed PrEP pills within the month does not alter risk of HIV acquisition. As a combined product, the DPP will also follow a 28-day regimen that includes 3 weeks (days 1–21) of co-formulated PrEP/COC pills followed by 1 week (days 22–28) of oral PrEP pills only. This allows for monthly bleeding in week 4, as COCs do, while maintaining protection against HIV. Formulating counseling recommendations for the DPP required weighing whether users could discard a DPP pack at week 4, in the event of multiple missed DPP pills in week 3, which aligns with COC practice and would lessen the time needed to use a back-up method for pregnancy prevention, but would confer additional supply and cost implications from disposing oral PrEP pills (which are more expensive than COCs and as such, increase the cost of DPP pills as well).

#### 2.2.2. Uptake

COC and oral PrEP guidance on uptake both recommend daily use during periods of increased risk of unintended pregnancy or HIV, even if the client does not have sex every day; use of a back-up method until full protective levels against pregnancy or HIV have been reached; taking one pill every day for the method to be effective and linking pill-taking to a daily activity to promote habitual use.

Guidance related to uptake differed on the time to reach protective levels of COC and oral PrEP. While COC guidance states that it takes up to 7 days to confer full protection against pregnancy, oral PrEP guidance documents varied: the 2017 WHO PrEP implementation tool and 2021 consolidated guidelines on HIV prevention state that it takes 7 consecutive days of PrEP use to build up protective levels against HIV (19, 30), yet the U.S. Centers for Disease Control and Prevention's (CDC) 2021 clinical practice guideline stipulates that 20 days of daily dosing is needed for maximum protection in cervicovaginal tissues (35). Because the research protocol for the DPP acceptability studies initially referenced CDC's data, the working group grappled with which source to utilize in its counseling recommendations, as it would impact how long clients would be counseled to use a back-up method at DPP initiation. Longer use of a back-up method could affect client acceptability of the DPP, particularly for COC users who are accustomed to using a back-up method for a shorter period.

Recommendations for uptake also reconciled instructions for use given that COC packs contain 21 days of COC pills followed by 7 days of non-hormonal pills, while oral PrEP tablets have no set order and are taken daily throughout the month. Because the non-hormonal pills in a COC pack are placebos, users may opt to discard them and start a new pack early. A key reason COC users do this is to avoid menstruation, which is a component of client self-care and satisfaction with the method (36). Clear counseling that week 4 of the DPP pack contains oral PrEP pills, which are not placebos and are needed to maintain protection against HIV, will be new to many COC users and critical to ensure correct use of the DPP.

#### 2.2.3. Side effects

COC and oral PrEP guidance both emphasize that side effects are typically not harmful, reduce over time, can often be self-managed and are not experienced by every user. They reinforce the importance of counseling clients on the most common side effects, how to manage or minimize them (e.g., by taking a pill the same time each day) and dispelling myths and misconceptions.

However, there is little overlap between types of side effects for COC and oral PrEP, requiring counseling messages for the DPP to outline potential side effects from each component rather than harmonizing them. Of particular importance in COC guidance is describing potential changes in bleeding patterns, which are commonly experienced with COC use but not with oral PrEP. Furthermore, time to resolution of side effects differs: PrEP side effects tend to dissipate after the first few weeks of use, while side effects for COCs may subside over the first few months. The potential for increased intensity of side effects with the DPP, given the co-formulation of oral PrEP and COC, is unknown, as is whether intensity of side effects could be further exacerbated by doubling up on missed doses.

#### 2.2.4. Discontinuation and switching

COC and oral PrEP guidance both underscore that the desire to use COC or PrEP can change over the life course, including due to perception of risk and side effects, and providers should employ informed choice counseling to discuss other available prevention options for women who want to discontinue or switch, emphasizing that they can do so at any time. Both oral PrEP and COCs have high discontinuation rates (37, 38). Counseling on the DPP requires clearly explaining how to manage the risks of HIV and unintended pregnancy when discontinuing or switching from the DPP to other prevention methods.

There are several scenarios related to discontinuation and switching on which a client may require counseling. Healthcare providers who offer the DPP will be trained to provide client-centered counseling to users who wish to discontinue the DPP. The counseling will ensure that clients understand their choices and are able to make decisions that fit their needs and lifestyle and will support them to achieve effective method switching (for FP, PrEP or both) if and when desired. Women who wish to become pregnant but still want to have protection against HIV may discontinue use of the DPP and can choose to continue use of oral PrEP or switch to another available HIV prevention option (e.g., condoms, PrEP ring or CAB for PrEP, where available). Women who no longer regard themselves at risk of HIV but do not wish to become pregnant can choose an alternative FP method. Women who want both FP and HIV prevention, but do not wish to use the DPP, can choose another combination of prevention methods that better suits their needs and preferences. Lastly, women who want neither FP nor HIV prevention can choose to use no method.

In most cases, a client can switch from the DPP to other FP and/or HIV prevention methods, including separate COC and/or oral PrEP products, right away. To ensure protection against HIV for clients who are stopping or switching, oral PrEP guidance suggests that providers recommend any of the following: take oral PrEP for 7 days after the last potential exposure before discontinuation; consider PEP if there is potential exposure before starting a new method and take an HIV test before restarting oral PrEP or starting a new HIV prevention method. Per COC guidance, providers should caution clients that their return to fertility is immediate in case of discontinuation. If a client wants to switch to another method and pregnancy can be reasonably ruled out using a checklist or a pregnancy test (26), the transition to a traditional COC or alternate contraceptive method can be expedited.

#### 2.2.5. Drug interactions

COC and oral PrEP guidance on drug interactions were the most straightforward to reconcile for the DPP among the prioritized counseling topics. The key message, drawn particularly from oral PrEP guidance and supported by published literature, is that PrEP and hormonal contraceptives can be safely and effectively taken simultaneously (39). Oral PrEP and COC have contraindications with different types of medicines, limiting the extent to which they could be integrated in counseling messages for the DPP. Contraindications for use of either oral PrEP or COC will be addressed as part of the medical eligibility determination when selecting a prevention method.

#### 2.2.6. Monitoring

There is essentially no overlap of monitoring requirements for COC and oral PrEP in counseling guidance. Most women can safely use COC and it can be initiated with no blood or other laboratory tests. A blood pressure test is recommended, but not required, prior to initiation of COC and annually thereafter. Clients are encouraged to revisit health providers annually and counseled to seek follow-up care if they are not satisfied with the method, cannot tolerate the side effects or experience symptoms associated with cardiovascular issues, but regular clinical monitoring is not required. Oral PrEP guidance is more complex; a negative HIV test is required prior to initiation and regularly thereafter (e.g., every three months), and depending on services offered and a client's profile, providers may also recommend creatinine screening, hepatitis B and/or C testing and STI screening or testing. (In 2022, the WHO released a technical brief on differentiating and simplifying PrEP delivery, which reduced complexity in required monitoring (40).) Developing monitoring recommendations for the DPP requires balancing these clinical requirements for oral PrEP with potential burden on clients, particularly for PrEP-naïve users who will be new to more extensive HIV monitoring, as well as health facility capacity to offer testing and screening services, which may be more limited in resource-constrained settings.

## 3. Actionable recommendations

Based on the analysis of COC and PrEP guidance outlined above, including discussion of outstanding questions and evidence gaps for the DPP, the working group developed the following counseling recommendations for the DPP. The recommendations provide a starting point for counseling on a novel MPT well in advance of product introduction, which can be adapted for and tested in clinical cross-over acceptability studies, and iterated upon based on findings. These messages are written for providers to share with clients after they have selected the DPP. They are intended to guide provider conversations with clients, recognizing that clients will have individual needs and questions that may require discussion beyond the messages included here. Additional messages will be needed for the initial FP and HIV prevention counseling session, so that users are aware of the DPP alongside a range of other prevention methods, but the development of these messages is outside the scope of this analysis.

### 3.1. Uptake (for new users or users who are restarting the DPP)

• Take one pill every day for the DPP to be effective until the pack is empty, even if you do not have sex every day.
• Each pack is a 28-day regimen. Days 1–21 contain COC and oral PrEP, and protect against pregnancy and HIV. Days 22–28 do not contain COC to allow for monthly bleeding; however, they do contain oral PrEP and pills should be taken to maintain HIV protection.
• Take the DPP for 7 consecutive days to reach protective levels against pregnancy and HIV. A back-up protection method should be used during this time.
  ○ COCs are protective against pregnancy right away if you start them within 5 days after the start of your monthly bleeding, and they take 7 days to confer full protection if taken at any other time during your menstrual cycle.
• It is common to have changes to your monthly bleeding when you use COCs. Common changes with the DPP may include irregular bleeding in the first few months, followed by lighter bleeding, shorter bleeding, spotting (dots of blood) and/or more regular bleeding (29).
• The DPP does not protect against other STIs. Use condoms for triple protection.
• Note to provider:
  ○ If a client wants to skip monthly bleeding and begin a new pack at the start of week 4, she can be counseled to do so and there are no foreseen risks. However, this is contingent on sufficient supply of refills.
  ○ If the client vomits after taking a DPP pill, she should follow the missed pills guidance.

### 3.2. Missed pills

• If you miss 1 pill one time in a month: take DPP as soon as you remember, even if it means taking 2 pills in one day. Do not take more than 2 pills in a day. Keep going with the pack. Any missed pills can increase your risk of pregnancy and HIV acquisition.
• If you miss 1 pill multiple times in a month or 2+ consecutive pills: take DPP as soon as you remember, even if it means taking 2 pills in one day. Do not take more than 2 pills in a day.
  ○ If 2+ consecutive pills are missed, you should only take the last missed pill as soon as possible, even if it means taking 2 pills in one day, and discard the other missed pills. Continue with the pack.
  ○ Option 1: Continue the pack through week 3 and then begin a new pack at the start of week 4. Note to provider: this is intended to recoup pregnancy protection faster and is contingent on a sufficient supply of refills.
  ○ Option 2: Continue through the end of the pack and use a back-up method (e.g., condoms) for pregnancy prevention for up to 3 weeks. Note to provider: 7 consecutive days of DPP pills containing COC are required to recoup pregnancy protection.
• Any missed pills can increase your risk of pregnancy and HIV acquisition. Missed pills in weeks 1 and 3 of the pack may further increase your risk of pregnancy.
  ○ Consider EC for pregnancy prevention if you have condomless sex within 5 days of 3+ missed pills.
  ○ Consider PEP for HIV prevention if you have condomless sex within 3 days of missed pills.
• Note to provider: If the client vomits within 2 hours after taking a DPP pill, she should take another pill from her pack as soon as possible, then continue with the pack. If vomiting or diarrhea continues for more than 2 days, she should follow 2+ missed pills guidance above.

### 3.3. Side effects

• You may experience side effects when you start using the DPP. Side effects are not signs of illness. They are typically mild and go away without treatment. Some women do not experience any side effects.
  ○ Common side effects with COCs include: headache, breast tenderness, weight change and possibly others. They usually lessen or stop within the first few months of use.
  ○ Common side effects with oral PrEP include: nausea, headache, abdominal cramping and vomiting. They usually lessen or stop within the first few weeks of use.
• It is common to have changes to your monthly bleeding when you use COCs. Common changes with the DPP may include irregular bleeding in the first few months, followed by lighter bleeding, shorter bleeding, spotting (dots of blood) and/or more regular bleeding.
• If you experience side effects, keep taking the DPP. Skipping pills increases risk of pregnancy and HIV acquisition, and can worsen some side effects. Try to take the DPP at the same time every day to minimize side effects. Try to link pill-taking to a daily activity to help you remember (e.g., with a meal, during your morning routine). Taking a pill at the same time each day can help reduce irregular bleeding and taking a pill with food can help avoid nausea.
• Speak to your healthcare provider if you have concerns about side effects or would like guidance on how to manage them. If you experience repeated headaches, you should speak to your provider.

### 3.4. Discontinuation/switching

• You may decide to discontinue use of the DPP or switch to another method of HIV prevention and/or contraception at any time. If you decide to discontinue use of the DPP, a provider can help you determine whether another prevention method is a better fit for your lifestyle and preferences.
• If you think that you have been exposed to HIV, you should continue taking the DPP for 7 days before discontinuing to ensure that you maintain protection during this period.
• If you discontinue use of the DPP, but want to be protected from HIV:
  ○ In most cases, you can begin using oral PrEP on its own or another biomedical HIV prevention method right away.
  ○ If there has been a lapse in DPP or PrEP use, a provider may recommend that you take an HIV test prior to starting a new HIV prevention method, even if your last routine HIV test was less than 3 months ago.
  ○ If you think you have been exposed to HIV after stopping use of the DPP, but before starting a new prevention HIV method, your provider may recommend PEP.
• If you discontinue use of the DPP, but do not want to become pregnant:
  ○ In most cases, you can begin using another contraceptive method right away. If you want to switch to another hormonal contraceptive method, a provider may ask you a few questions to be reasonably certain that you are not pregnant or have other contraindications to a method beforehand. Note to provider: See pregnancy checklist on page 463 of WHO's FP Handbook (26).

### 3.5. Drug interactions

• There are no drug-drug interactions from combining oral PrEP and COC in the DPP. Use of hormonal contraceptives while taking PrEP is safe and effective.
• Certain medications are not recommended for women interested in the DPP due to their contraindication with COC, including but not limited to certain anticonvulsants, lamotrigine, rifampicin and rifabutin. Note to provider: Refer to the WHO FP Handbook and the WHO Medical Eligibility for Contraceptive Use for the full list of COC contraindications (26, 41).
• Certain medications are not recommended for women interested in the DPP due to their contraindication with oral PrEP, including but not limited to adefovir and certain medications that reduce renal function. Note to provider: Refer to the CDC's PrEP guidelines (2021) for the full list of PrEP contraindications (35).

### 3.6. Monitoring

• You will need to get an HIV test prior to initiating or restarting the DPP, and every 3 months during DPP use (Table 2). Your provider may also ask you to take an HIV test one month after initiation. It is possible that these HIV tests could be HIV self-tests.
  ○ If you think you have had a recent HIV exposure (e.g., within the past 72 hours), your provider may offer you PEP and transition you to the DPP after completing PEP and HIV testing.
  ○ Your provider may also recommend EC if you have had condomless sex within the past 5 days.
• A very small percentage of people will not be eligible for the DPP because oral PrEP is not recommended for users with reduced kidney function. Your provider may ask you to do creatinine screening within the first few months of DPP initiation (e.g., if you are 30+ years old or have comorbidities) and may recommend additional screening every 6–12 months.
• Your provider may recommend testing for hepatitis B and C at DPP initiation, and hepatitis C annually thereafter. Your provider may also recommend vaccination and/or additional testing in the future. You can initiate the DPP before your hepatitis B and C results are available.
• Your provider may recommend STI screening or testing every 3–6 months.
• Your provider may recommend you take a blood pressure test prior to initiating the DPP and annually thereafter.

**Table 2:** DPP monitoring recommendations.

DPP Monitoring Recommendations						
	Initiation	3 Months	6 Months	9 Months	12 Months	Notes
HIV Testing	✓	✓	✓	✓	✓	Recommended every 3 months.
Creatinine Screening	✓		✓		✓	Optional for those <30 years old without kidney-related co-morbidities. Recommended once within the first few months of DPP initiation for those 30+ years old without co-morbidities. Recommended every 6–12 months for individuals with co-morbidities.
Blood Pressure Testing	✓				✓	Recommended at initiation and then on an annual basis.
Hepatitis B Testing	✓					Recommended at initiation. Vaccination or additional testing may be recommended in the future.
Hepatitis C Testing	✓				✓	Recommended at initiation and then on an annual basis. Vaccination or additional testing may be recommended in the future.
STI Screening or Testing	✓		✓		✓	Recommended every 3–6 months.

## 4. Discussion

Comprehensive counseling by providers is one of the core tenets of quality SRH/HIV services. In FP literature, high-quality contraceptive counseling—which is client-centered and prioritizes voluntarism and informed choice—is associated with contraceptive use and method continuation (42–44). Similarly, for oral PrEP, provider-initiated counseling that includes information on perceived risk as well as strategies for managing side effects and adherence supports continued use (45, 46). Through counseling, providers serve as a crucial access point to women's awareness of the available prevention methods, their key characteristics, how to use their selected method correctly and how to manage associated side effects.

Yet there are significant and documented barriers to providers' successful delivery of contraceptive counseling (47, 48). In some cases, providers' knowledge of methods is inadequate or incorrect (42). Counseling can be influenced by providers' own experience with contraception and their biases about what methods are most suitable for women (e.g., for young women, unmarried women or women living with HIV) (49, 50). For example, a commonly cited bias is that AGYW are not good candidates for COC or oral PrEP due to their inability to take a daily pill and that for women living with HIV, the use of COC increases pill burden (i.e., needing to take two pills versus one) (49, 51). Notably, formative research with providers conducted to inform DPP acceptability studies found that providers described the DPP as having the potential to lessen the burden of taking two separate pills for COC and oral PrEP as well as to reduce the frequency and increase the efficiency of clinic visits (17).

In settings where FP and HIV services have been integrated, and where counseling on novel MPTs like the DPP will be required, additional barriers exist. Providers are often short on time and when new methods are introduced, their time is further stretched to attend trainings, incorporate new counseling messages and documentation requirements and to manage women's questions and concerns (49, 52). Among FP providers, a lack of training for HIV testing as well as to screen for and/or provide oral PrEP can contribute to a lack of confidence in discussing PrEP with women and feelings that it is “out of scope” (8, 53).

Reconciling counseling messages for missed pills was challenging due to divergent guidance for COC and oral PrEP. However, even within FP literature, client instructions on missed pills are not well understood. Research shows that more than 60% of oral contraceptive users know what to do when one pill is missed but far fewer know what to do when two or more pills are missed (54). To remedy confusion, providers are recommended to give simple, straightforward instructions—both verbally and written, including the use of graphics—as well as a contact for questions in the event of a missed pill (55). Taking this advice into consideration, the working group endeavored to develop counseling recommendations that are simple, concise and can be easily understood and acted upon by providers and users alike. For future MPT delivery forms, in particular longer-acting and/or provider-administered products, some counseling topics, like missed doses, may be irrelevant or easier to reconcile.

End-user research with potential DPP users, providers and male partners found that side effects are one of the largest concerns of both prospective DPP clients and providers (56), which could have implications for user acceptability and provider willingness to offer the DPP. For both COC and oral PrEP, the provision of information on side effects and how to manage them improves outcomes and continued use (46, 57, 58). Counseling users to understand potential changes to bleeding patterns is key to user satisfaction and continuation with other FP methods (59, 60), and by extension, is expected to be critical to effective use of the DPP. According to the Method Information Index, which is a measure of quality contraceptive counseling, women should be informed about the possibility of side effects with their selected method, how to manage them if they occur and alternate FP methods, including other oral contraceptives (61). Counseling messages on the latter will need to be developed to situate the DPP within the broader contraceptive method mix.

## 5. Conclusion

Developing recommendations for the DPP as a novel MPT posed unique challenges, with implications for efficacy, cost and user and provider comprehension and burden. Incorporating DPP counseling recommendations into clinical cross-over acceptability studies is an opportunity to receive feedback in real-time from both providers and users on their clarity and utility. Such feedback allows for iterative revision to increase the ease of delivery for providers as well as user comprehension and efficacy (62). Fine-tuning the counseling messages so that women can use the method correctly and confidently is critically important for eventual scale and commercialization of the DPP.
